# The Relative Merits of Ether and Chloroform as Anesthetics

**Published:** 1884-04

**Authors:** J. W. Parkinson


### THE RELATIVE MERITS OF ETHER AND CHLOROFORM AS ANESTHETICS.*

BY J. W. PARKINSON, M. D.

[Pacific Medical and Surgical Journal.]

* * * Having assumed, and perhaps proved, that an apparatus is essential for the comfortable and steady administration of ether, while chloroform can be given without any special contrivance, the possession of such apparatus gives to neither anesthetic superiority in point of time. Excluding the quantity of the drug required to produce the effect, anesthesia by ether can be induced with some practice and attention to detail in an average time of two minutes; whilst as far as my personal experience goes the range is from 40 seconds to 10 minutes. This result is barely equaled by chloroform, for whilst the average time is about the same, the range is limited in one direction and extended in the other.

Few will be prepared to deny that the induction of anesthesia with chloroform in less time than one and a half or two minutes would be attended with greatly increased risk to the subject; whilst the period of ten minutes is not infrequently exceeded in fruitless efforts to produce insensibility.

The preliminary stage of excitement, which is more marked in

* Conclusions of a paper read before the Sacramento Society for Medical Improvement, ether than chloroform administration, can usually be considerably shortened by concentration of the vapor.

When the use of ether became tolerably general, and before efficient inhalers had been constructed, the persistence of this stage, which often became alarmingly violent, led to the erroneous supposition that for operations requiring absolute muscular relaxation, as reduction of dislocations, fractures, etc., ether was inadmissible. Practical experience and improved surgical appliances have fully disproved this, and there is no stage of anesthesia which cannot now be as readily reached and maintained by this agent as by chloroform.

RELATIVE SAFETY AND DANGER—In estimating the safety of an anesthetic, we may first assume its general applicability, and that no special contra-indications exist. Granting that ether and chloroform are equally admissible in any case, to determine their relative safety it will be necessary for a moment to consider their physiological action.

Both drugs, if we accept the theory, produce the anesthetic state by causing "a decrease and cessation of that molecular motion," on which we agree all organic processes depend. As the phenomena of active life depend primarily on the healthy functional activity of the two great centers, circulatory and respiratory, so it follows that any reagent which causes paralysis of one, and in a much greater degree of both, must be used with extreme caution. Both chloroform and ether, as a rule, lower the vascular tension and decrease the cardiac impulse. This holds good in the majority of cases after the patient has passed the preliminary stage of excitement. While universally true in the case of chloroform, there are many exceptions to it where ether is the agent employed.

In numbers of instances carefully observed, and in experiments on animals, it has been proved beyond question that the vascular tension and cardiac impulse continue unchanged throughout the period of insensibility, and in some instances are positively increased.

Briefly, the ultimate effect of these agents, if pushed to toxic extremes, is in the case of ether to produce arrest primarily of the respiratory and next of the circulatory center; in that of chloroform, first of the circulatory and next of the respiratory center, but very frequently of both simultaneously.

Ether acts partly by paralyzing the pulmonary circulation, and also directly on the lung tissue. Chloroform is a direct paralyzer of the heart itself, affecting its motor apparatus.

Coze, in 1849, demonstrated that if a few drops were injected into the jugular vein of an animal, paralysis of the right heart followed immediately. If injected into the lungs, paralysis of the left heart ensued. In subsequent experiments a like result was obtained in equal time, though the vagi had been divided. These facts are well shown in the following table:

| | Chloroform. | Ether. |
|---|---|---|
| Time required to produce complete stoppage of pulmonary circulation | 75 seconds | 270 seconds |
| Amount of anesthetic vapor employed | 50 cc. | 500 cc. |
| Quantity of air necessary to re-establish circulation in lung | 600 cc. | 200 cc. |
| Time occupied in restoring circulation | 720 seconds | 180 seconds |
| Heart's impulses before artificial respiration | 18 | 24 |
| Heart's impulses when circulation was stopped | 4 | 6½ |

The rapid failure of the circulatory system, the small quantity of the anesthetic capable of producing the result, the quantity of air necessary to restore that circulation, and the time occupied in its restoration in the case of chloroform, bear a very unfavorable relation to those in which ether had been employed.

The history of accidents occurring during anesthesia, as well as those of suspended animation due to other causes, teaches how far readier it is to arouse the dormant respiratory function than to affect the heart. And while the due performance of one insures the fulfillment of the other, the means generally at hand are only applicable to the former. From this it seems rational to infer that the agent which first sensibly affects respiration before the vascular tension has reached a stage beyond reasonable hope of restoration, and during whose use simultaneous arrest of both functions is almost unknown, offers a wide margin of safety.

In maintaining the position that under no circumstances should chloroform be used where ether can be substituted without increasing the risk, there is one contra-indication which should not be forgotten. The existence of pulmonary trouble especially affecting the larger tubes and producing excessive mucoid secretion, renders the employment of this agent decidedly dangerous.

In the healthy subject one of the first effects produced by ether inhalation is hypersecretion of the glands and follicles lining the respiratory tract; and if persistent, the accumulation of such secretion in the larynx and fauces becomes a source of apprehension. When the mucous membrane is abnormal and a considerable amount of secretion already exists, the vapor acting on the previously irritated member so increases the accumulation as to render prolonged anesthesia an impossibility. At least one death in the list of those from ether is directly attributable to neglect of this fact.

By what may be more than mere coincidence, the presence of malignant disease seems to materially add to the risks of ether administration; but the data are far too incomplete to permit of a reliable estimate being formed.

In the department of ophthalmic surgery, from some of the phenomena which it induces, ether is objectionable. That flushing of the face and neck, so comfortable a sign to the anesthetist, is to the oculist fraught with unpleasant forebodings. The increased vascular tension and capillary injection, of which it is the evidence, cause turgescence of the hemic channels supplying the orbit and its contents. Hemorrhage within any part of the eyeball is undesirable, and where the iris is wounded during the operation chloroform offers a decided superiority.

Besides the considerations of physiological or pathological import, there are others of expediency which of necessity compel us by force of circumstances to act in a manner contrary to our wishes and judgment if more favorably situated. We have assumed that the efficient and safe administration of ether demands an apparatus of approved construction; whereas it is clearly demonstrated that for chloroform nothing more complicated than a piece of cloth is desirable. In country practice, alone and unassisted, often at a distance of many miles from help, perhaps summoned in the middle of the night, the practitioner, who must be anesthetist, operator and assistant in the case, had better trust his folded handkerchief to a bystander than any inhaler, however effective. Again, where long journeys have to be made on horseback, the addition of any article that can be dispensed with is inexpedient.

When ether is being administered for the first time to any person, and often on subsequent occasions, sufficient assistance to control the struggles during the few preliminary respirations is essential; and where this is not procurable the attempt must end in failure more or less complete.

Many children will take chloroform almost readily, and the apparent immunity which early life enjoys from its toxic effect makes it for short operations the most desirable agent. Child-birth seems to confer a like degree of safety. The cases of fatality due directly to the action of the drug are very rare; and as its employment here is more frequently to mitigate the severity than to completely abolish pain, chloroform still remains "par excellence" the anesthetic.

When operating by artificial light, the great inflammability of ether and its rapid diffusion in the air of an apartment must be borne in mind. Several explosions, accompanied by injury more or less severe to those present, have occurred from neglect or ignorance of this fact; and unless the light is so protected, or at such a distance as to insure safety to all concerned, chloroform had better be substituted.

In forming a conclusion as to the relative merits of the drugs under discussion, statistics must be referred to; and here, as in other cases, they are far from satisfactory. Attempts have been made in this and other countries to tabulate cases where death has occurred, but the subjects are as a rule so imperfectly reported, and so much that is vital to the result omitted, that many are not fairly attributable directly to the anesthetic. No doubt a very large number of deaths during administration are never published, those only finding their way into print which have occurred in hospitals, whilst the mortality in private practice remains untold. As Professor Lyman quaintly puts it, "Occasionally an elderly physician alludes in a cautious manner to a case of which he was cognizant long years ago in a remote quarter of the earth."

Dr. Andrews, of Chicago, was the first to collect a large number of cases. His figures are:

Ether—92,815 administrations; 4 deaths; 1 in 23,204.

Chloroform—117,078 administrations; 43 deaths; 1 in 2,732.

Coles, of Virginia, gives: Chloroform—152,620 administrations; 53 deaths; 1 in 2,873.

B. W. Richardson: Chloroform—35,165 administrations; 11 deaths; 1 in 3,196. He had, however, seen it used 15,000 times before he met the first fatal case.

Billroth had given it 12,500 times, with a like result.

Ker, Royal Infirmary, Edinburgh, in 10 years gave 36,500 administrations, with 1 death.

Bardeleben gives 30,000 cases, and no death.

The result of these and some additional figures is:

Ether—99,255 administrations; 6 deaths; 1 in 16,542.

Chloroform—492,233 administrations; 84 deaths; 1 in 5,800.

Morgan, of Dublin, gave as the result of Andrews' and Richardson's tables:

Ether—92,815 administrations; 4 deaths; 1 in 23,204.

Chloroform—152,260 administrations; 53 deaths; 1 in 2,873.

With such widely different results from chloroform as 1 death in

30,500 and 1 in 2,873, and there is no reason to doubt the accuracy of either statement, these figures must be cautiously received.

That a larger number of deaths, inhalation for inhalation, have occurred with chloroform than with ether, is beyond dispute. That as the use of ether is becoming more general we fail to hear of an increase in the death rate, or even of the same number of fatalities, while an occasional death is still reported from chloroform, cannot be denied.

That ether and chloroform are equally effective as anesthetics; that where ether is employed the onset of alarming symptoms is as a rule preceded by ample warning, while in chloroform often none is given till a fatal result has actually occurred; that chloroform is a powerful depressant of the vascular system and direct paralyzer of the heart; while ether increases the vascular tension, affects the respiration primarily and the heart secondarily, are propositions beyond refutation.

We may, as our result, adopt the following conclusions:

1st. That ether is as efficient an anesthetic as chloroform. 2d. That there are fewer cases in which its use is contra-indicated. 3d. That it is a safer anesthetic in the hands of the most experienced, and by inference correspondingly in an increased ratio with those more or less unskilled. 4th. That the use of chloroform with our present knowledge and experience, in preference to ether, where no contra-indication to the latter can be shown, is adding materially to the risk of the patient and the responsibility of the administrator.

